# Two new species of the genus *Teredorus* Hancock, 1906 (Orthoptera, Tetrigidae) from China, with a key to the species of the genus

**DOI:** 10.3897/zookeys.431.8002

**Published:** 2014-08-05

**Authors:** Wei-An Deng, Chao-Liang Lei, Zhe-Min Zheng

**Affiliations:** 1 Hubei Insect Resource Utilization and Sustainable Pest Management Key Laboratory, College of Plant Science and Technology, Huazhong Agricultural University, Wuhan 430070, China; 2 School of Chemistry and Bioengineering, Hechi University, Yizhou 546300, Guangxi, China; 3 Institute of Zoology, Shaanxi Normal University, Xi’an 710062, China

**Keywords:** Caelifera, Tetrigoidea, Taxonomy, new species, biology, China

## Abstract

Taxonomy of a tetrigid genus *Teredorus* Hancock is reviewed. Two new species, *Teredorus parvipulvillus* **sp. n.** and *Teredorus hunanensis* **sp. n.** are described from China and an updated identification key to all known species of the genus is given, as well as brief comments on phylogenetic relationships, biology and ecology.

## Introduction

The genus *Teredorus* was erected by Hancock in 1906, with *Teredorus stenofrons* Hancock as its type species; it was collected from Peru in South America. Hancock (1915) described *Teredorus carmichaeli* Hancock, 1915 and *Teredorus frontalis* Hancock, 1915 from India. Since Hancock (1915), there are twenty-four species of this genus that have been found in China and India, i.e., *Teredorus longipulvillus* Zheng, 1988, *Teredorus bhattacharyi* Shishodia, 1991, *Teredorus truncates* Shishodia, 1991, *Teredorus hainanensis* Zheng, 1993, *Teredorus wuyishanensis* Zheng, 1993, *Teredorus bashanensis* Zheng, 1993, *Teredorus guizhouensis* Zheng, 1993, *Teredorus prominemarginis* Zheng & Jiang, 1993, *Teredorus albimarginus* Zheng & Zhou, 1996, *Teredorus camurimarginus* Zheng, 1998, *Teredorus bidentatus* Zheng, Huo & Zhang, 2000, *Teredorus flatimarginus* Zheng & Liang, 2000, *Teredorus ebenotus* Zheng & Li, 2001, *Teredorus fujianensis* Zheng & Li, 2001, *Teredorus guangxiensis* Zheng, Shi & Luo, 2003, *Teredorus xishuiensis* Zheng, Li & Shi, 2003, *Teredorus flavistrial* Zheng, 2006, *Teredorus bipulvillus* Zheng, 2006, *Teredorus eurylobatus* Zheng, Shi & Mao, 2010, *Teredorus taibeiensis* Zheng & Xu, 2010, *Teredorus brachinota* Zheng & Xu, 2010, *Teredorus brachinotoides* Zheng, Ou & Lin, 2012, *Teredorus choui* Zheng, Ou & Lin, 2012, *Teredorus nigropennis* Deng, Zheng & Lu, 2013. Additionally, Zheng and Xu (2010) reviewed the genus *Teredorus* from China and adjacent countries, *Systolederus grasveli* Günther, 1939 was transferred into the genus *Teredorus* Hancock and *Teredorus truncates* Shishodia, 1991 was transferred into the genus *Systolederus* Bolivar by them. Thus, to date, the genus includes 27 known species worldwide, distributed mainly in South America, China, India and Nepal.

The genus *Teredorus* Hancock is considered as a member of the subfamily Tetriginae based on the following characters: the filiform antennae; width of frontal costa narrower than first segment of antenna; lateral lobes of pronotum turned downwards, posterior angles rounded; the first segment of posterior tarsi equal to or slightly longer than the third one.

The identification of the genus *Teredorus* is problematic due to the absence of an identification key at the species level, so the original descriptions of the genus *Teredorus* are brief and imprecise. In this paper, the clarification of the taxonomic status of the genus *Teredorus* is revised and the utility of the characters used to diagnose is determined. In addition, *Teredorus parvipulvillus* sp. n. and *Teredorus hunanensis* sp. n. are described from China and an updated key to the species of *Teredorus* is given, as well as brief comments on phylogenetic relationships, biology and ecology.

## Material and methods

Specimens examined are deposited in the following institutions: Institute of Zoology, Shaanxi Normal University, Xi’an, China (IZSNU); School of Chemistry and Bioengineering, Hechi University, Yizhou, China (SCBHU).

Photographs were taken with an Olympus digital camera with a series of images montaged using the program CombineZ5.3 (Hadley 2006).

Morphological terminology and measurement landmarks method followed those of Zheng (2005) and Deng et al. (2007). Descriptions of the species are mostly based on female specimens because many Tetriginae males are usually difficult to discriminate and thus the identification is usually done by association with females collected at the same time and place. Measurements are given in millimetres (mm). The specimens measured correspond to the material designated as holotypes, allotypes and paratypes.

## Taxonomy

### Teredorus

Taxon classificationAnimaliaOrthopteraTetrigidae

Hancock, 1906

Teredorus Hancock 1906: 51; Hancock 1915: 109; Kirby 1910: 30; Bruner 1910: 118; Shishodia 1991: 70–71; Blackith 1992: 181; Liang and Zheng 1998: 130; Zheng 2005: 219; Zheng 2006: 21–22; Deng et al. 2007: 201–202; Zheng and Xu 2010: 14.

#### Type species.

*Teredorus stenofrons* Hancock, 1906, Southern America, by original designation.

#### Redescription.

Size small or medium. Colour varying from ashy to brown and dark brown. body smooth, interspersed with granules.

Head a little or not elevated above the pronotal surface; vertex very strongly narrowed toward the front drawing the eyes together and in front forming a triangular shape, median carinula distinct and not advanced in front of the eyes; face slightly oblique; frontal costa bifurcate just behind lateral ocelli, elevated and compressed between antennae, sinuate in front, moderately sulcate. Antennae filiform, located below the eyes. Eyes more or less globose or pear-shaped, a little or not elevated above the pronotal surface, drawing antero-medially to each other; lateral ocelli situated below middle of eyes.

Pronotum anteriorly truncate, dorsum smoothly granulate, somewhat flattened, but subcylindrical, all the carinae low, median carina depressed or indistinct forward in front shoulders, posteriorly moderately distinct; prozonal carinae obsolete; humeral angles obtuse; pronotal process extend beyond apex of hind femora; lateral lobes of pronotum turned downwards, posterior angles rounded, posterior margin of each lateral lobe with two concavities. Elytra elongate, ovate with acuminate apex. Wings extended beyond the pronotal apex. Fore femora elongate, a little broadened, very finely serrated; middle femora elongate, broadened, bicarinate, margins finely serrated; hind femora elongate, a little crassate, margins finely serrated; first and third segment of hind tarsi equal in length, first pulvilli a little smaller than the second and third, second and third pulvilli equal in length.

#### Differential diagnosis.

The morphology of *Teredorus* is quite homogeneous, and this genus can be easily distinguished from other genera of the subfamily Tetriginae by vertex very strongly narrowed toward the front drawing the eyes together and in front forming a triangular shape.

#### Key to the species of *Teredorus* Hancock

**Table:** 

1	Tegmina and wings absent; hind process of pronotum just reaching the middle of hind femur; hind margin of lateral lobe of pronotum only with one concavity. Distribution in India	*Teredorus bhattacharyi* Shishodia, 1991
–	Tegmina and wings present; hind process of pronotum reaching or surpassing the apex of hind femur; hind margin of lateral lobe of pronotum with two concavities	2
2	First segment of hind tarsi with two pulvilli. Distribution in China	*Teredorus bipulvillus* Zheng, 2006
–	First segment of hind tarsi with three pulvilli	3
3	Inner margin of hind tibia without internal spine; second pulvillus of posterior tarsus degenerated, extremely smaller than first and third. Distribution in China	*Teredorus parvipulvillus* sp. n.
–	Inner margin of hind tibia with a row of internal spines; three pulvilli of posterior tarsus normal	4
4	Pronotum short, reaching or just surpassing the top of hind femora	5
–	Pronotum elongate, extending far beyond the top of hind femora	6
5	Frontal ridge straight before lateral ocellus; hind wings not reaching apex of hind process of pronotum; inner side of hind femur black; lower inside of hind femur black, outside pale brown; sternum of abdomen black. Distribution in China	*Teredorus brachinota* Zheng & Xu, 2010
–	Front al ridge slightly concave before lateral ocellus; hind wings reaching apex of hind process of pronotum; inner side of hind femur yellowish brown, basal part black; lower inside of hind femur yellowish brown, with two black spots, outside black; sternum of abdomen yellowish brown. Distribution in China	*Teredorus brachinotoides* Zheng, Ou & Lin, 2012
6	Head not exerted above upper level of pronotum	7
–	Head distinctly exerted above upper level of pronotum	13
7	Hind wings developed, surpassing apex of posterior process of pronotum	8
–	Hind wings not or just reaching apex of posterior process of pronotum	11
8	Vertex visible before eyes in lateral view, vertex and frontal ridge forming rounded; upper margin of pronotum slightly undulating before shoulders and straight behind shoulders in profile	9
–	Vertex not visible before eyes in lateral view; upper margin of pronotum arched or straight in profile	10
9	Frontal ridge a little concave between lateral ocelli in profile; posterior process of pronotum reaching one third of hind tibia; width of mid femur equal to tegmina; first segment of posterior tarsus equal to third in length, three pulvilli of first segment of posterior tarsus equal in length. Distribution in China	*Teredorus hunanensis* sp. n.
–	Frontal ridge straight before lateral ocellus in profile; posterior process of pronotum reaching apex of hind tibia; width of mid femur narrower than width of tegmina; length of first segment of posterior tarsus longer than third segment, third pulvillus of first segment of hind tarsi longer than the first and second pulvilli. Distribution in China	*Teredorus flavistrial* Zheng, 2006
10	Body smaller, length of pronotum ♂ 10–11 mm, ♀ 13-14 mm; upper margin of pronotum arched in profile; width of mid femur equal to width of tegmina. Distribution in S. America	*Teredorus stenofrons* Hancock, 1907
–	Body larger, length of pronotum. ♂ 15–16 mm, ♀ 17–18 mm; upper margin of pronotum straight in profile; width of mid femur narrower than width of tegmina. Distribution in China	*Teredorus guangxiensis* Zheng, Shi & Luo, 2003
11	Upper and lower margins of fore and mid femora undulating; third pulvillus of first segment of hind tarsi longer than the first and second pulvilli. Distribution in China	*Teredorus longipulvillus* Zheng, 1988
–	Upper and lower margins of fore and mid femora straight; three pulvilli of first segment of posterior tarsus equal in length	12
12	Body smaller, Length of pronotum: ♀ 13.5–14.0 mm; hind process of pronotum reaching middle of hind tibia; length of upper valvulae 2.8× its width. Distribution in China	*Teredorus nigropennis* Deng, Zheng & Lu, 2013
–	Body larger, Length of pronotum: ♀ 17–18 mm; hind process of pronotum reaching apex of hind tibia; length of upper valvulae 4× its width. Distribution in China, India and Nepal	*Teredorus carmichaeli* Hancock, 1915
13	Antennae inserted between lower margins of eyes	14
–	Antennae inserted under lower margins of eyes	16
14	Width of mid femur wider than width of tegmina; lower outer side of hind femur black. Distribution in China	*Teredorus albimarginus* Zheng & Zhou, 1996
–	Width of mid femur narrower than or equal to width of tegmina; lower outer side of hind femur brown	15
15	Posterior process of pronotum reaching two third of hind tibia; width of mid femur narrower than width of tegmina; middle of posterior margin of subgenital fig of female triangularly projecting. Distribution in China	*Teredorus hainanensis* Zheng, 1993
–	Posterior process of pronotum reaching one third of hind tibia; width of mid femur equal to width of tegmina; posterior margin of subgenital fig of female straight. Distribution in China	*Teredorus flatimarginus* Zheng & Liang, 2000
16	Mid keel of pronotum interrupted before shoulders; complete after shoulders; lateral keels of prozona interrupted. Distribution in India	*Teredorus gravelyi* (Günther, 1939)
–	Mid keel of pronotum entire; lateral keels of prozona entire	17
17	With abbreviated carinae between shoulders	18
–	Without abbreviated carinae between shoulders	19
18	Upper margin of pronotum straight in profile; lateral keels of pronotal prozona inconspicuous; posterior margin of female subgenital fig slightly concave in the middle Distribution in China	*Teredorus camurimarginus* Zheng, 1998
–	Upper margin of pronotum slightly convex before shoulders and undulating behind shoulders in profile; lateral keels of pronotal prozona slightly constricted backward; posterior margin of subgenital fig of female slightly triangularly projecting in the middle. Distribution in China	*Teredorus fujianensis* Zheng & Li, 2001
19	Upper margin of pronotum undulating before shoulders in profile. Distribution in China	*Teredorus wuyishanensis* Zheng, 1993
–	Upper margin of pronotum staight in profile	20
20	Middle of posterior margin of subgenital fig of female concave. Distribution in China	*Teredorus xishuiensis* Zheng, Li & Shi, 2003
–	Posterior margin of female subgenital fig straight or with two teeth or with three teeth or triangularly projecting	21
21	Disc of pronotum black; posterior margin of subgenital fig of female straight. Distribution in China	*Teredorus ebenotus* Zheng & Li, 2001
–	Disc of pronotum not black; posterior margin of subgenital fig of female with two teeth or with three teeth or triangularly projecting	22
22	Posterior margin of subgenital fig of female with two teeth. Distribution in China	*Teredorus bidentatus* Zheng, Huo & Zhang, 2000
–	Posterior margin of subgenital fig of female with three teeth or triangularly projecting	23
23	posterior margin of female subgenital fig with three teeth	24
–	Middle of posterior margin of female subgenital fig triangularly projecting	25
24	Antennae 15-segmented; hind wings reaching apex of posterior process of pronotum. Distribution in China	*Teredorus guizhouensis* Zheng, 1993
–	Antennae 16-segmented; hind wings developed, surpassing apex of posterior process of pronotum. Distribution in China	*Teredorus eurylobatus* Zheng, Shi & Mao, 2010
25	Hind wings surpassing apex of posterior process of pronotum. Distribution in China	*Teredorus bashanensis* Zheng, 1993
–	Hind wings reaching apex of posterior process of pronotum	26
26	Width of mid femur narrower than width of tegmina; mid-keel of dorsal side of hind femur dentate. Distribution in China	*Teredorus taibeiensis* Zheng & Xu, 2010
–	Width of mid femur wider than or equal to width of tegmina; mid-keel of dorsal side of hind femur smooth	27
27	Hind process reaching apex of hind tibia; width of mid femur wider than width of tegmina. Distribution in India and Nepal	*Teredorus frontalis* Hancock, 1915
–	Hind process reaching middle of hind tibia; width of mid femur equal to width of tegmina	28
28	In lateral view, vertex and frontal ridge forming a rounded angle, visible before eyes; middle of posterior margin of subgenital fig of female triangularly projecting. Distribution in China	*Teredorus choui* Zheng, Ou & Lin, 2012
–	In lateral view, vertex not visible before eyes; posterior margin of subgenital fig of female with sharp angular protuberance. Distribution in China	*Teredorus prominemarginis* Zheng & Jiang, 1993

### Teredorus parvipulvillus sp. n.

Taxon classificationAnimaliaOrthopteraTetrigidae

http://zoobank.org/4458D6D2-167F-439E-8203-D1F3771E09EE

Figs 1 –2

#### Female.

**Figure 1.**
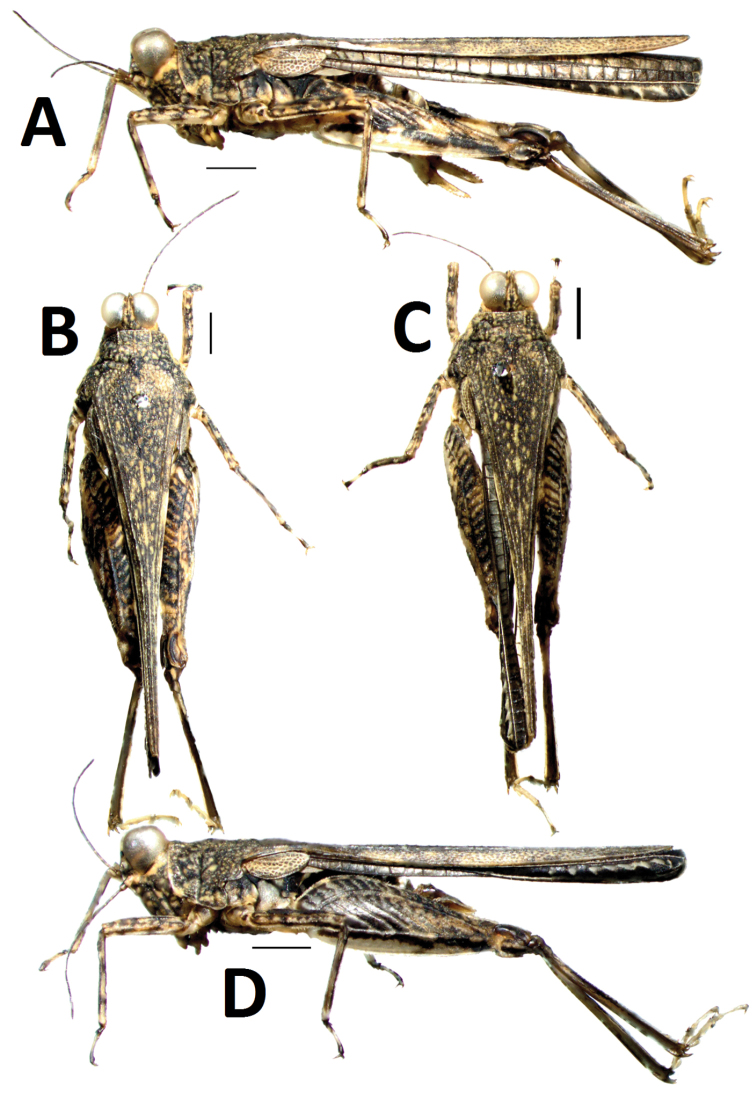
*Teredorus parvipulvillus* sp. n. **A** female, lateral view of body **B** female, dorsal view of body **C** male, dorsal view of body **D** male, lateral view of body. (scale bar = 1mm).

**Figure 2.**
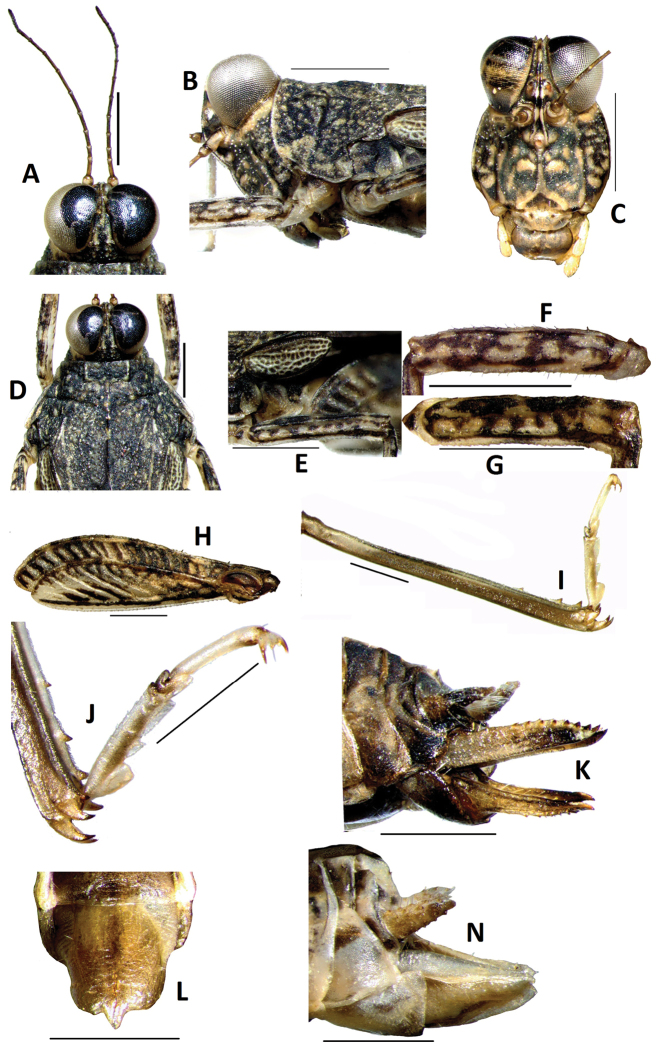
*Teredorus parvipulvillus* sp. n. **A** dorsal view of head **B** lateral view of head **C** frontal view of head **D** dorsal view of head and pronotum **E** lateral view of tegmina and mid femur **F** lateral view of fore femur **G** lateral view of mid femur **H** lateral view of hind femur **I** lateral view of hind tibia and tarsus **J** lateral view of hind tarsus **K** lateral view of ovipositor of female **L** ventral view of subgenital fig of female **N** lateral view of subgenital fig of male. (scale bar = 1mm).

Size small, slender. Length of body (from vertex to apex of hind process) 3.8 times as its width (between posterior angles of lateral lobes of pronotum), head distinctly exerted above upper level of pronotum (Fig. 1A). In dorsal view, vertex strongly contracted forward drawing the eyes very near together (Fig. 2A, D), not exserted before eyes, midkeel distinct, extended to occiput; vertex not visible before eyes in lateral view, frontal ridge straight before lateral ocellus, arc-protruding between antennae, longitudinal furrow narrower than width of 1st segment of antennae. Antenna filiform, 15-segmented, inserted between lower margin of eyes (Fig. 2B), mid segments 4-7 times as long as wide. Eyes elevated above the pronotum (Fig. 2B), globular in shape, lateral ocelli placed on slightly lower than middle of anterior margins of eyes (Fig. 2C).

Disc of pronotum smooth, with numerous small granules, mid keel of pronotum entire, upper margin of pronotum slightly undulating before shoulders and straight behind shoulders in profile (Fig. 1A). Anterior margin of pronotum straight, lateral keels of prozona short and parallel (Fig. 2D), humeral angle obtuse, without abbreviated carinae between shoulders. Posterior process of pronotum narrow, long cone-shaped, surpassing apex of hind femur and reaching apex of hind tibia (Fig. 1A, B). Lateral lobes of pronotum turned downwards, posterior angles rounded, posterior margin of each lateral lobe with two concavities. Visible part of tegmina ovate (Fig. 2E), apex narrowly rounded, with length 2.5 times its width. Hind wings developed, reaching and slightly surpassing apex of posterior process of pronotum (Fig. 1A). Upper and lower margins of fore femur and mid femur nearly straight (Fig. 2F, G), width of mid femur narrower than width of tegmina (1: 1.4) (Fig. 2E). Hind femur stubby (Fig. 2H), with length 3.3 times its width, mid keel of dorsal and ventral side of hindfemur dentate, antegenicular right angle and genicular denticles acute angle. Outer side of hind tibia with two to three spines, inner side without spine (Fig. 2I). Length of first segment of posterior tarsus longer than third, first pulvillus and third normal, equal in length, apex abtuse; second pulvillus degenerated and very small (Fig. 2J), apex acute. Ovipositor narrow and long, length of upper valvulae 4.2 times its width, upper and lower valvulae with slender saw-like teeth(Fig. 2K). Length of subgenital fig equal to width, middle of posterior margin of subgenital fig triangularly projecting (Fig. 2L).

*Colouration*. Disc of pronotum and head blackish-brown with numerous light marks (Fig. 1A, B). Antennae dark brown (Fig. 2A). Hind wings black. Tergites and sternites black. Outer side of hind femora dark, with white marks; inner side black. Hind tibiae black, with brown at base.

#### Male

(Fig. 1C, D). Similar to female, but smaller and narrower. Subgenital fig short, cone-shaped (Fig. 2N).

#### Measurements.

Length of body ♂5.5–6.0 mm, ♀ 7.0–7.5 mm; length of pronotum ♂8.0–8.5 mm, ♀ 9.0–9.5 mm; length of hind femur ♂3.5–4.0 mm, ♀ 4.0–4.5 mm.

Holotype female and allotype male, China, Guangxi, Ningming, Aidian, 21°52'N, 107°03'E, 320m alt, 22 July. 2013, collected by Wei-An DENG, IZSNU. Paratypes: Same data, ten male; six females, collected by Wei-An DENG, IZSNU (3♂2♀), SCBHU (7♂4♀).

#### Diagnosis.

This species can be easily distinguished from other species of the genus by inner side of hind tibia without spine; second pulvillus of posterior tarsus degenerated, distinctly smaller than first and third. It is only similar to *Teredorus ebenotus* Zheng & Li, 2001, from which it differs in: antenna inserted between lower margin of eyes; upper margin of pronotum slightly undulating before shoulders and straight behind shoulders in profile; posterior process of pronotum reaching apex of hind tibia; width of mid femur distinctly narrower than tegmina. *Teredorus ebenotus* Zheng & Li, 2001 exhibits antenna inserted below lower margin of eyes; upper margin of pronotum straight in profile; posterior process of pronotum reaching middle of hind tibia; width of mid femur equal to tegmina.

#### Etymology.

The new species' name is derived from Latin *parv* and *pulvillus*, meaning second pulvillus of posterior tarsus degenerated and very small.

#### Habitat.

The new species lives in moist stony on the border of streams in tropical rainforests.

#### Distribution.

China (Guangxi).

### Teredorus hunanensis sp. n.

Taxon classificationAnimaliaOrthopteraTetrigidae

http://zoobank.org/564AAF62-3BB8-4324-BEF2-3D916E448019

Figs 3 –4

#### Female.

Size small, slender. Length of body (from vertex to apex of hind process) 3.2 times as its width (between posterior angles of lateral lobes of pronotum), head not exerted above upper level of pronotum (Fig. 4B). In dorsal view, vertex strongly contracted forward drawing the eyes very near together(Fig. 4D), not exserted before eyes, midkeel distinct, extended to occiput; vertex visible before eyes in lateral view, vertex and frontal ridge forming rounded, frontal ridge slight concave between lateral ocelli, arc-protruding between antennae, longitudinal furrow narrower than width of 1st segment of antennae. Antenna filiform, 15-segmented, inserted below lower margin of eyes (Fig. 4B, C), mid segments 5-6 times as long as wide. Eyes globose, lateral ocelli placed on slightly lower than middle of anterior margins of eyes(Fig. 4C).

**Figure 3.**
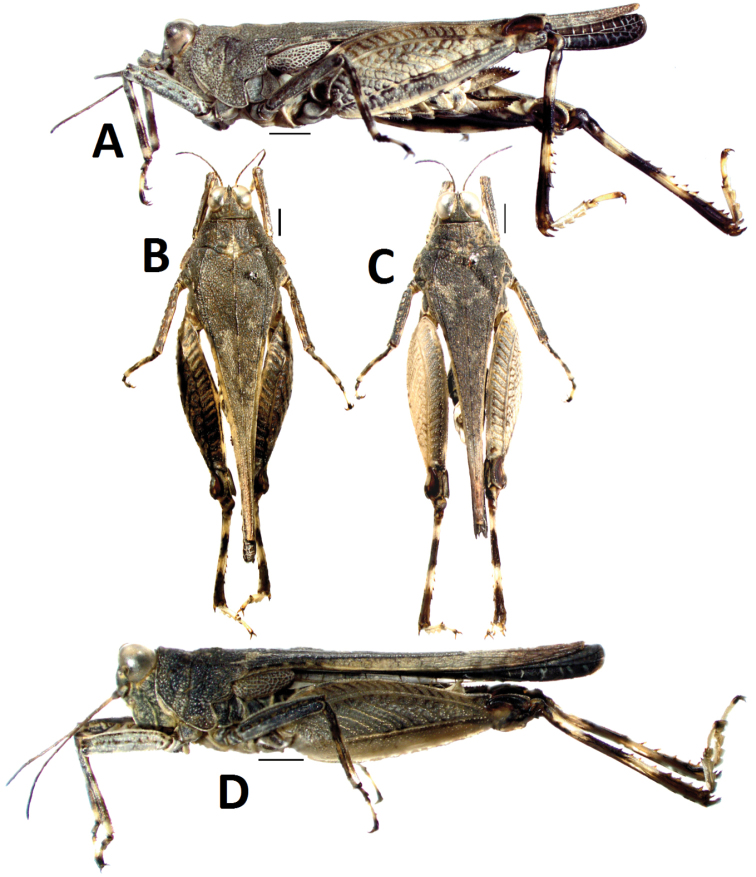
*Teredorus hunanensis* sp. n. **A** female, lateral view of body **B** female, dorsal view of body **C** male, dorsal view of body **D** male, lateral view of body. (scale bar = 1mm).

**Figure 4.**
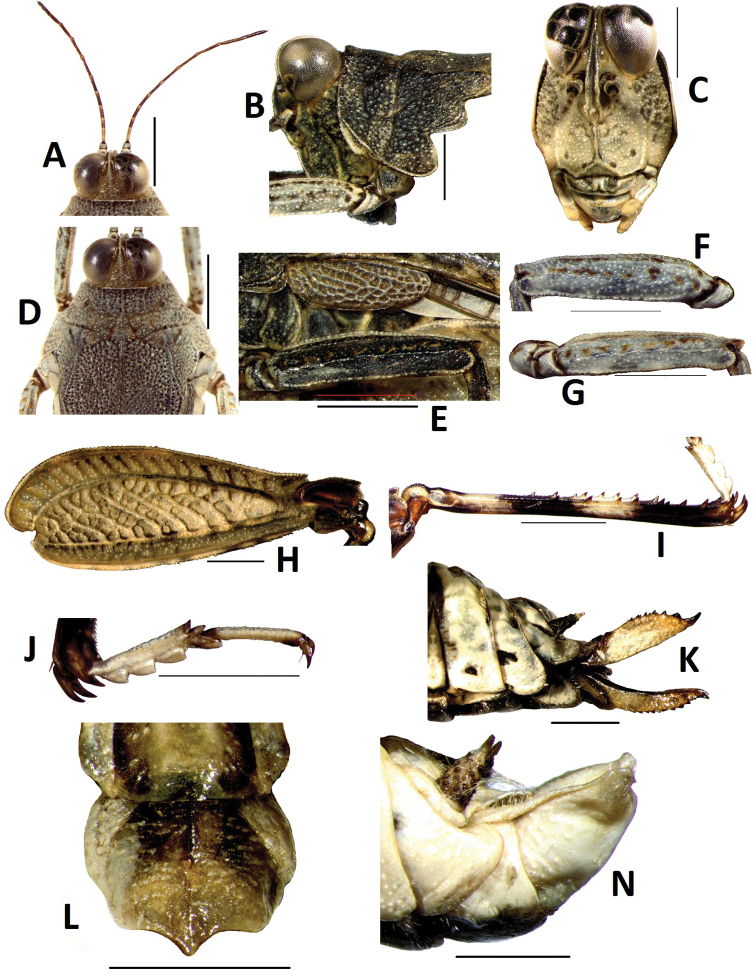
*Teredorus hunanensis* sp. n. **A** dorsal view of head **B** lateral view of head **C** frontal view of head **D** dorsal view of head and pronotum **E** lateral view of tegmina and mid femur **F** lateral view of fore femur **G** lateral view of mid femur **H** lateral view of hind femur **I** lateral view of hind tibia **J** lateral view of hind tarsus **K** lateral view of ovipositor of female **L** ventral view of subgenital fig of female **N** lateral view of subgenital fig of male. (scale bar = 1mm).

Disc of pronotum smooth, with numerous small granules, mid keel of pronotum entire (Fig. 3B), upper margin of pronotum slightly undulating before shoulders and straight behind shoulders in profile (Fig. 3A). Anterior margin of pronotum straight, lateral keels of prozona unconspicuous, parallel (Fig. 4D), humeral angle obtuse, without abbreviated carinae between shoulders. Posterior process of pronotum narrow, long cone-shaped, surpassing apex of hind femur and reaching one third of hind tibia (Fig. 3A). Lateral lobes of pronotum turned downwards, posterior angles rounded, posterior margin of each lateral lobe with two concavities. Visible part of tegmina ovate (Fig. 4E), apex narrowly rounded, with length 2.8 times its width. Hind wings developed, reaching and slightly surpassing apex of posterior process of pronotum (Fig. 4A). Upper and lower margins of fore femur and mid femur straight (Fig. 4F, G), width of mid femur equal to tegmina. Hind femur stubby (Fig. 4H), with length 2.8 times its width, mid keel of dorsal and ventral side of hindfemur dentate, antegenicular right angle and genicular denticles acute angle. Outer side of hind tibia with five to six spines, inner side with four to six spines (Fig. 4I). First segment of posterior tarsus equal to third in length, three pulvilli equal in length, apices of all pulvilli abtuse (Fig. 4J). Ovipositor narrow and long, length of upper valvulae 3 times its width, upper and lower valvulae with slender saw-like teeth (Fig. 4K). Length of subgenital fig equal to width, middle of posterior margin of subgenital fig triangularly projecting (Fig. 4L).

*Colouration*. Body dark brown. Hind wings black. Fore and mid femora brown, with two black rings in the middle, first segment of tarsi black, apex of second segment black. Hind femora dark brown or brown, inner side black. Hind tibiae black, with two yellowish brown rings in the middle (Fig. 3A).

Male (Fig. 3C, D). Similar to female, but smaller and narrower. Subgenital fig short, cone-shaped (Fig. 4N).

#### Measurements.

Length of body ♂7.5–8.0 mm, ♀ 9.5–10.0 mm; length of pronotum ♂9.5–10.0 mm, ♀ 11.5–12.0 mm; length of hind femur ♂5.0–5.5 mm, ♀ 6.5–7.0 mm.

Holotype female and allotype male, China, Hunan, Yuanling, Jiemuxi National Nature Reserve, 28°45'N, 110°26'E, 650m alt, 06 Aug. 2013, collected by Yu-Hua DONG, IZSNU. Paratypes: Same data, seven male; eight females, collected by Yu-Hua DONG and Yan-Lan Feng, IZSNU (3♂2♀), SCBHU (4♂6♀).

#### Diagnosis.

This species is similar to *Teredorus flavistrial* Zheng, 2006, from which it differs in: frontal ridge slight concave between lateral ocelli in profile; posterior process of pronotum reaching one third of hind tibia; width of mid femur equal to tegmina; first segment of posterior tarsus equal to third in length, three pulvilli of first segment of posterior tarsus equal in length. *Teredorus flavistrial* Zheng, 2006, exhibits frontal ridge straight before lateral ocellus in profile; posterior process of pronotum reaching apex of hind tibia; width of mid femur narrower than width of tegmina; length of first segment of posterior tarsus longer than third segment, third pulvillus of first segment of hind tarsi longer than the first and second pulvilli.

#### Etymology.

The new species was named after the type locality, Hunan, China.

#### Habitat.

The new species lives in moist stony on the border of streams in rainforests.

#### Distribution.

China (Hunan).

## Discussion

**Figure 5.**
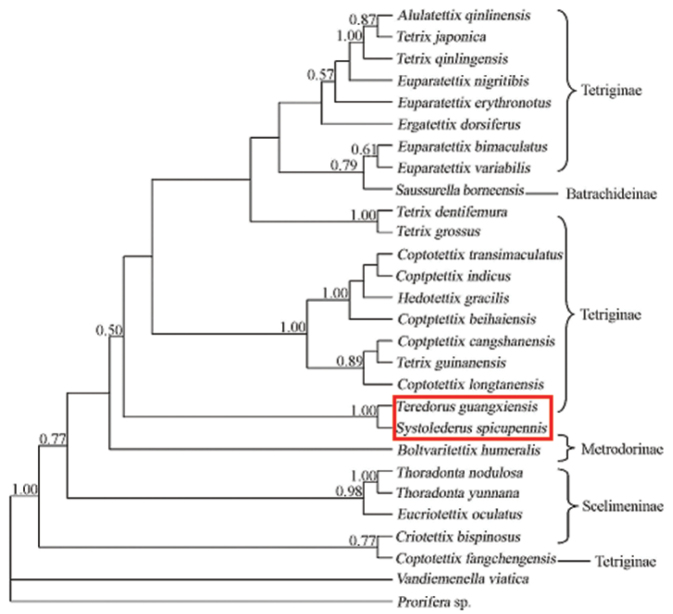
Bayesian phylogenetic hypothesis generated using molecular characters (mitochondrial cytochrome c oxidase subunit iv sequence data), assuming GTR + I + G model Values above the branches indicate Bayesian posterior probability, and bootstrap values are listed above nodes which had>50%. (drawing by Fang et al.).

The original description of the genus *Teredorus* Hancock is based on characters from the external morphology exclusively. The morphology of *Teredorus* is quite homogeneous, and the species can be differentiated mostly by having vertex very strongly narrowed toward the front drawing the eyes together and in front forming a triangular shape. Recent molecular studies, based on *Teredorus guangxiensis* cytochrome c oxidase subunit I gene (Fang et al. 2010) showed *Teredorus* Hancock, 1906 (subfamily Tetriginae) to be closely related to the genus *Systolederus* Bolivar, 1887 (subfamily Metrodorinae) (Fig. 5), but Fang et al. did not further comment why these two genera are related in morphology. The external morphology of the species of *Teredorus* is very similar to that of the species of *Systolederus*, in spite of being included in different subfamilies, these two genera share vertex very strongly narrowed toward the front drawing the eyes together and in front forming a triangular shape. *Teredorus* is mainly characterized by having lateral lobes of pronotum turned downwards, posterior angles rounded, *Systolederus* exhibits lateral lobes of pronotum produced outwards, with truncate posterior angles. Externally, its segregation is based on the relative shape of posterior angles of lateral lobes of pronotum, which is a diagnostic character to effectively separate Metrodorinae from Tetriginae. Accordingly, *Teredorus* and *Systolederus* can combine to the same genus, but need to be further studied.

The genus shows a striking disjunct distribution, with its type species known from Peru in South America and other members of the genus from China, India and Nepal in Asia. We infer that *Teredorus* may be widely distributed all over the world, but they have not been collected and studied at present in most of continents, e.g. Africa, Europe, American Continent, etc.

According to the diagnosis of the Tetriginae (Zheng 2005), *Teredorus parvipulvillus* sp. n. and *Teredorus hunanensis* sp. n. clearly belong to this subfamily. We place two new species in *Teredorus* based on lateral lobes of pronotum turned downwards, posterior angles rounded. Additional characters support this placement, as pronotum generally not produced above head in front, antenna filiform, frontal costa moderately forked between antennae, not forming a frontal scutellum, its width narrower than the basic segment of antenna, usually separated by a sulcus.

Many species of the genus *Teredorus* are usually associated with, but not limited to, moist environments, living along the moist stony on the border of streams. Some have suggested that with the pronotum subcylindrical and smooth, the often flattened front and median limbs are used like paddles for swimming. Their diet consists mostly of algae growing on the moist stony surface, along with lichens and other forms of humus. They generally overwinter as adults. *Teredorus* are likely to disappear if environments become polluted or disturbed by human beings.

## Supplementary Material

XML Treatment for Teredorus

XML Treatment for Teredorus parvipulvillus

XML Treatment for Teredorus hunanensis
